# Supplementary material for: Cooperation of LIM domain‐binding 2 (LDB2) with EGR in the pathogenesis of schizophrenia
Source: EMBO Mol Med. 2021 Mar 3;13(4):e12574. doi: 10.15252/emmm.202012574 (PMC8033514; doi:10.15252/emmm.202012574)
Supplement: Supplementary file 1 — Appendix [file EMMM-13-e12574-s001.pdf]

Appendix File, Ohnishi T., *et al.*

Table of Contents

- *Appendix Tables*

1. Appendix Table S1
2. Appendix Table S2
3. Appendix Table S3
4. Appendix Table S4

- *Appendix Figures*

5. Appendix Figure S1
6. Appendix Figure S2
7. Appendix Figure S3
8. Appendix Figure S4

Appendix Table S1. Summary of behavioral analyses of *Ldb2* KO mouse

Test	Results	Validity as a schizophrenia model
Open field test	Hyperactivity*	Positive symptom-related.
Home cage activity	Hyperactivity	Positive symptom-related.
Elevated plus maze	Normal	
Light dark transition	Normal	
Tail suspension	Normal	
Prepulse inhibition	No reduction	
Social interaction (Encounter method)	No reduction	
Rotarod test	Normal in motor coordination	
Y maze test	Alternation reduced (tendency)	Working memory deficits.
Hot plate test	Normal	
Fear conditioning (contextual test)	Deduced freezing*	Cognitive deficits.
Fear conditioning (cued test)	Deduced freezing*	Cognitive deficits.
Barnes maze	Normal	
8-Arm radial maze	Normal	
Morris water maze	Moving speed reduced day-by-day	Abnormal mood control
Locomotor activity by dopamine agonist	Exggerated locomotor actity by methyphenidate	Positive symptom-related
Locomotor activity by NMDA antagonist	Exggerated locomotor actity by MK-801	Positive symptom-related
Neurogenesis in the dentate gyrus	Significantly reduced	Yes

* These phenotypes were ameliorated by the treatment with antipchychotics or lithium salt.

Appendix Table S2. Summary of the sequencing data used for the ChIP-seq analysis

Cells	Read category	Accession	# Raw reads	# Reads after trimming	# Uniquely mapped reads	# Peaks called by MACS2
Neuroshere	input	DRA010493 (this study)	25,917,604	25,910,873	22,615,308	14,545
	ChIP	DRA010493 (this study)	15,742,990	15,623,377	13,304,886	
ECC1	input	SRR577960, SRR577961	69,068,747	65,833,683	50,654,757	12,661
	ChIP	SRR577989, SRR577990	61,928,846	57,924,494	45,639,854	
H1ES	input	SRR577840, SRR577841	83,500,187	71,558,442	58,119,276	12,727
	ChIP	SRR351691, SRR351692	58,088,393	49,544,038	39,135,817	
HCT116	input	SRR577956, SRR577957	68,496,387	62,739,879	50,210,480	10,144
	ChIP	SRR577901, SRR577902	46,895,186	40,520,593	32,029,919	
K562	input	SRR578001, SRR578002, SRR578003, SRR578004	176,829,940	145,526,245	115,798,395	59,385
	ChIP	SRR351661, SRR351662	70,572,446	60,927,720	48,494,406	

Appendix Table S3. Genes with peaks overlapping at upstream regions or 5'UTRs between LDB2 and EGR1-ChIP

LOC643837	FAM20B	APLNR	C12orf47	PRTG	MPRIP	FZR1	HK2	SLC5A3	PARL	ARHGAP26	C7orf10
LOC100130417	QSOX1	SSRP1	C12orf51	NEDD4	MED9	HMG20B	POLE4	CHAF1B	ABCC5	PRELID2	GLI3
SAMD11	LHX4	UBE2L6	PTPN11	TCF12	RASD1	PIP5K1C	DNAH6	HLCS	AP2M1	SH3RF2	STK17A
C1orf170	JA429802	AK096335	PLBD2	FAM63B	PEMT	APBA3	C2orf89	PIGP	ABCF3	TCERG1	AX747737
HES4	LOC284648	YPEL4	C12orf49	RNF111	RAI1	SH3GL1	TMSB10	DYRK1A	ALG3	PPP2R2B	POLD2
AGRN	LAMC1	CLP1	FBXW8	BNIP2	SMCR5	HDGFRP2	KCMF1	BRWD1-IT2	CAMK2N2	AK056780	CAMK2B
AK310350	DKFZP564C19	ZDHHC5	KSR2	TPM1	DRG2	PLIN5	TCF7L1	HMGN1	PSMD2	CSNK1A1	ZMIZ2
C1orf159	SMG7	C11orf31	RFC5	AK055197	LLGL1	TNFAIP8L1	USP39	MX1	EIF4G1	PPARGC1B	PPIA
TNFRSF18	ARPC5	VPS37C	WSB2	LACTB	FAM83G	C19orf10	ATOH8	C2CD2	POLR2H	NDST1	PURB
UBE2J2	C1orf21	VWCE	TAOK3	RPS27L	EPN2	TRNA_Gly	ST3GAL5	PDE9A	CHRD	MYOZ3	NACAD
PUSL1	C1orf27	PPP1R32	RAB35	APH1B	RNF112	PLIN3	PTCD3	NDUFV3	TRA2B	C5orf62	HUS1
DVL1	RGS13	MIR4488	PXN	USP3	SLC47A1	HSD11B1L	KDM3A	PKNOX1	EIF4A2	GM2A	C7orf57
AURKAIP1	RGS2	DAGLA	MSI1	PPIB	ALDH3A2	FUT6	ZNF514	U2AF1	BCL6	SLC36A1	UPP1
VWA1	TROVE2	C11orf9	TRIAP1	TRIP4	ULK2	VMAC	BC016831	SIK1	LPP-AS2	AX747215	VWC2
ATAD3A	CDC73	FADS3	LOC100506668	OAZ2	AKAP10	RFX2	CIAO1	PDXK	CCDC50	GALNT10	GRB10
LOC643988	DENND1B	AHNAK	MLEC	RBPMS2	TMEM11	MLLT1	KANSL3	CSTB	MB21D2	HAND1	COBL
GNB1	DDX59	MTA2	UNC119B	PIF1	VTN	ALKBH7	CNNM4	AGPAT3	HRASLS	CNOT8	ZNF713
TMEM52	CAMSAP2	ROM1	SPPL3	PLEKHO2	RAB34	GTF2F1	SEMA4C	TRAPPC10	OPA1	ADRA1B	MRPS17
SKI	KIF21B	UBXN1	HNF1A-AS1	SPG21	ERAL1	SLC25A23	COX5B	PWP2	BC038368	PWWP2A	CCT6P3
PEX10	LAD1	POLR2G	KDM2B	CLPX	ABHD15	TRIP10	ACTR1B	PFKL	HES1	FBLL1	VKORC1L1
PANK4	PHLDA3	WDR74	ORAI1	IGDCC3	TP53I13	ZNF557	TMEM131	LRRC3	LOC100131551	PANK3	TPST1
HES5	TIMM17A	CHRM1	TMEM120B	MAP2K1	ANKRD13B	INSR	VWA3B	FAM207A	CPN2	RANBP17	RABGEF1
FLJ42875	RNPEP	C11orf95	HPD	SMAD6	BLMH	TRAPPC5	C2orf55	ADARB1	LSG1	FGF18	AUTS2
LRRC47	KDM5B	RCOR2	PSMD9	SMAD3	SUZ12P	SNAPC2	MITD1	COL18A1	XXYLT1	FBXW11	FZD9
RPL22	ADORA1	NAA40	B3GNT4	CORO2B	ATAD5	CTXN1	REV1	PCBP3	MUC20	STK10	TBL2
HES3	LOC730227	VEGFB	RSRC2	MIR548H4	RAB11FIP4	FBN3	LONRF2	COL6A1	OSTalpha	UBTD2	VPS37D
GPR153	ATP2B4	PPP1R14B	DENR	UACA	MIR193A	CERS4	C2orf29	COL6A2	RNF168	SH3PXD2B	WBSCR22
ACOT7	SNRPE	ESRRA	VPS37B	PKM2	C17orf79	ANGPTL4	MAP4K4	MCM3AP-AS1	FBXO45	NEURL1B	ELN
KLHL21	SOX13	SF1	ABCB9	PARP6	LRRC37B	ADAMTS10	LOC150568	DIP2A	LRRC33	ERGIC1	LIMK1
PHF13	PPP1R15B	ATG2A	OGFOD2	NEO1	RHOT1	ZNF558	LOC100506421	S100B	PIGX	C5orf41	RFC2
DNAJC11	AX746920	ZNHIT2	SETD8	CD276	RHBDL3	FBXL12	POU3F3	CECR6	LOC100507086	NKX2-5	CLIP2
CAMTA1	RBBP5	POLA2	RILPL2	ARID3B	MIR632	OLFM2	TGFBRAP1	TUBA8	CPLX1	BOD1	HIP1
VAMP3	DSTYK	TIGD3	DDX55	CSK	CDK5R1	DNMT1	UXS1	CLTCL1	FGFRL1	MSX2	RHBDD2
PER3	TMCC2	SLC25A45	GTF2H3	CPLX3	MYO1D	MIR1181	LIMS1	TBX1	CTBP1	ARL10	POR
PARK7	KLHDC8A	NEAT1	ATP6V0A2	ULK3	TMEM98	KRI1	RANBP2	RANBP1	KIAA1530	CLTB	TMEM120A
GPR157	MIR135B	MALAT1	ZNF664-FAM1	COX5A	LIG3	ILF3	APLN	KLHL22	AX748388	EIF4E1B	YWHAG
H6PD	NUCKS1	EHBP1L1	UBC	UBE2O2	NLE1	CARM1	BCL2L11	PI4KA	FAM53A	TSPAN17	PTPN12
SPSB1	C1orf186	MAP3K11	UBB	WDR61	RASL10B	SMARCA4	MERTK	CRKL	SLBP	UIMC1	MAGI2-AS3
TMEM201	EIF2D	DQ587981	AX721264	CRABP1	GGNBP2	TSPAN16	TMEM87B	MGC16703	FGFR3	ZNF346	GNAI1
BC038541	PLXNA2	CFL1	RIMBP2	IREB2	SRCIN1	RAB3D	TTL	UBE2L3	WHSC1	MXD3	KIAA1324L
CLSTN1	DIEXF	MUS81	RAN	MORF4L1	MLLT6	EPOR	SLC20A1	YDJC	NAT8L	GRK6	DMTF1
CTNNBIP1	KCNH1	EFEMP2	ULK1	FAH	CISD3	PRKCSH	CKAP2L	MIR130B	NOP14	DBN1	C7orf23
UBE4B	RCOR3	CCDC85B	PUS1	ARNT2	PCGF2	CNN1	ACTR3	YPEL1	GRK4	N4BP3	TP53TG1
KIF1B	SLC30A1	DRAP1	EP400	FAM108C1	PIP4K2B	MAN2B1	EN1	MAPK1	FAM86EP	ZFP2	ABCB4
PGD	NEK2	PACS1	EP400NL	KIAA1199	RPL23	DHPS	TMEM37	ZDHHC8P1	STX18	BC040901	DBF4
CASZ1	PPP2R5A	CD248	LOC100507055	MESDC1	LASP1	C19orf43	PTPN4	AK096976	AK056081	TBC1D9B	GTPBP10
TARDBP	TATDN3	RIN1	POLE	MEX3B	RPL19	HOOK2	TMEM185B	DQ596074	MSX1	CNOT6	CDK14
FBXO6	FLVCR1	B3GNT1	AK055957	EFTUD1	MIEN1	JUNB	LOC84931	LRP5L	CRMP1	SCGB3A1	FZD1
C1orf187	KCTD3	DPP3	ZNF140	CPEB1	IKZF3	PRDX2	MKI67IP	ADRBK2	LOC285484	ZFP62	MTERF
MTHFR	IARS2	RCE1	ZNF10	HOMER2	ORMDL3	MAST1	BIN1	AK055980	KIAA0232	TRIM41	ANKIB1
PLOD1	MARK1	C11orf86	GJA3	BTBD1	PSMD3	NACC1	HS6ST1	MN1	GRPEL1	BC016291	PEX1
MFN2	MIA3	KDM2A	IFT88	ZNF592	NR1D1	STX10	MZT2B	TTC28	FLJ36777	DUSP22	TFPI2
TMEM51	TLR5	ANKRD13D	AK093279	AKAP13	MSL1	IER2	AX746725	ZNRF3	SH3TC1	AK023629	SGCE
EFHD2	TP53BP2	POLD4	ZDHHC20	DET1	CASC3	CACNA1A	FAM168B	KREMEN1	ACOX3	WRNIP1	PPP1R9A
CASP9	DEGS1	CLCF1	TNFRSF19	ACAN	KRT37	ZSWIM4	LYPD1	EMID1	5S_rRNA	NQO2	DYNC1I1
DNAJC16	ENAH	RAD9A	MIPEP	ABHD2	FKBP10	PRKACA	CCNT2	GAS2L1	RAB28	BPHL	LMTK2
DDI2	SRP9	RPS6KB2	BC043582	KIF7	NT5C3L	DNAJB1	RAB3GAP1	NIPSNAP1	LAP3	TUBB2A	BRI3
FLJ37453	C1orf55	TMEM134	CENPJ	ZNF710	KLHL11	NOTCH3	UBXN4	HORMAD2	LCORL	LOC100507194	NPTX2
FLJ00313	H3F3B	GSTP1	NUPL1	CIB1	CNP	BRD4	DARS	GATSL3	SLIT2	TUBB2B	TRRAP
RCC2	PARP1	NUDT8	WASF3	IQGAP1	DNAJC7	AKAP8L	CXCR4	TBC1D10A	GPR125	AK096219	ZSCAN21
ARHGEF10L	CDC42BPA	FAM86C2P	USP12	CRTC3	ATP6V0A1	RAB8A	SPOPL	RNF215	SEL1L3	PXDC1	ZNF3
MRTO4	WNT9A	SUV420H1	MTIF3	MAN2A2	COASY	KLF2	GTDC1	TCN2	C4orf52	PRPF4B	COPS6
CAMK2N1	MRPL55	PPP6R3	POMP	SV2B	RPL27	SLC35E1	ACVR2A	SLC35E4	RBPJ	AK092451	AP4M1
PINK1	GUK1	IGHMBP2	SLC7A1	SLCO3A1	RND2	C19orf42	EPC2	SELM	STIM2	FARS2	GPC2
HP1BP3	TRIM17	CCND1	UBL3	FAM174B	DHX8	TMEM38A	FMNL2	RNF185	PCDH7	NRN1	GATS
ALPL	ABCB10	FGF19	KATNAL1	RGMA	ETV4	MYO9B	ARL6IP6	PATZ1	RELL1	RREB1	MEPCE
HSPG2	TAF5L	CTTN	USPL1	IGF1R	MEOX1	NR2F6	RPRM	Em:AC005003.	PGM2	TMEM14C	PPP1R35
ZBTB40	COG2	DHCR7	EEF1DP3	AF020763	MPP3	ANO8	KCNJ3	DRG1	KLHL5	TMEM14B	ACTL6B
KDM1A	EGLN1	NADSYN1	PDS5B	LRRC28	G6PC3	FAM125A	AK128708	EIF4ENIF1	UGDH	TMEM170B	GNB2
LUZP1	KCNK1	RNF121	SMAD9	MEF2A	C17orf53	SLC27A1	ACVR1C	PRR14L	BC040333	HIVEP1	POP7
HNRNPR	C1orf31	INPPL1	MRPS31	CHSY1	ATXN7L3	GLT25D1	ACVR1	DEPDC5	ATP8A1	GFOD1	EPHB4
GALE	IRF2BP2	PHOX2A	SLC25A15	SNRPA1	RUNDC3A	KCNN1	STK39	HMGXB4	SLAIN2	SIRT5	SERPINE1
CLIC4	B3GALNT2	FCHSD2	TRNA_Glu	AXIN1	SLC25A39	RAB3A	METTL5	HMOX1	OCIAD2	RANBP9	VGF
MAN1C1	GPR137B	ARHGEF17	KBTBD6	FAM195A	FAM171A2	JUND	AK127400	MCM5	USP46	JARID2	PLOD3
C1orf135	ERO1LB	RELT	NAA16	RHOT2	GPATCH8	SSBP4	LOC440925	RBFOX2	RASL11B	DTNBP1	CUX1
PAQR7	ACTN2	FAM168A	C13orf15	FBXL16	FZD2	ELL	SP5	TXN2	CHIC2	GMPR	LOC100289561
PAFAH2	FMN2	PPME1	AKAP11	HAGHL	DBF4B	UBA52	AK023515	MPST	NMU	RBM24	ORAI2
ZNF593	FH	LOC100507050	GTF2F2	NARFL	ADAM11	TMEM59L	GAD1	KCTD17	REST	TPMT	LRWD1
SH3BGRL3	CHML	WNT11	CPB2	SOX8	C1QL1	COMP	HAT1	SSTR3	LPHN3	DEK	DNAJC2
LIN28A	ZNF238	AK125821	LRRC63	BC084558	HEXIM1	GATAD2A	RAPGEF4	CARD10	LOC550112	AK092541	LOC723809
RPS6KA1	ADSS	LRRC32	LRCH1	CACNA1H	LOC100133991	CCNE1	GPR155	CDC42EP1	UTP3	ID4	LOC100216546
ARID1A	FAM36A	TSKU	ITM2B	UBE2I	MAP3K14	TSHZ3	LOC401022	SH3BP1	MOB1B	SOX4	LOC100216545
SLC9A1	HNRNPU	CAPN5	EBPL	BAIAP3	MAPT	ZNF507	LOC375295	H1F0	ADAMTS3	GMNN	SRPK2
TMEM222	KIF26B	PAK1	KPNA3	UNKL	RPRML	GPATCH1	DFNB59	GCAT	ANKRD17	LRRC16A	PUS7
MAP3K6	SCCPDH	RSF1	SPRYD7	C16orf91	KPNB1	LRP3	PLEKHA3	MIR658	CXCL3	HCG11	NAMPT
GPR3	SH3BP5L	INTS4	TRIM13	CRAMP1L	TBKBP1	LOC80054	NEUROD1	MICALL1	PARM1	HMGN4	PRKAR2B
AHDC1	PFKP	NDUFC2-KCTI	MIR4703	MAPK8IP3	MRPL10	CEBPG	NCKAP1	MAFF	G3BP2	ZSCAN23	BCAP29
PPP1R8	KLF6	GAB2	WDFY2	GFER	CALCOCO2	CHST8	ZNF804A	LOC400927	USO1	LOC401242	NRCAM
DNAJC8	IL15RA	LOC100506233	ALG11	NPW	GIP	KCTD15	HIBCH	KDELR3	NUP54	GABBR1	ZNF277
PHACTR4	RBM17	TMEM126A	VPS36	SLC9A3R2	IGF2BP1	WTIP	INPP1	JOSD1	SCARB2	PPP1R11	IFRD1
YTHDF2	CELF2	SYTL2	PCDH17	MIR3180-5	SPOP	ZNF302	TMEM194B	GTPBP1	SH3KBP1	ABCF1	TMEM168
TMEM200B	CAMK1D	TMEM135	COMMD6	CASKIN1	SAMD14	ZNF181	NAB1	SUN2	CCNI	PPP1R10	MDFIC
SRSF4	SEPHS1	RAB38	UCHL3	ECI1	PPP1R9B	GRAMD1A	STAT1	CBX6	CCNG2	PPP1R18	ST7-AS1
MECR	TRDMT1	NAALAD2	KCTD12	CCNF	SGCA	SCN1B	MYO1B	CBX7	CNOT6L	NRM	CTTNBP2
PTPRU	PTPLA	C11orf75	FBXL3	NTN3	XYLT2	LOC100128675	OBFC2A	SYNGR1	FRAS1	FLOT1	KCND2
PTP4A2	STAM	C11orf54	SLAIN1	PDPK1	LUC7L3	FXYD7	PGAP1	SMCR7L	BMP2K	IER3	FAM3C
KHDRBS1	ARL5B	AMOTL1	SPRY2	LOC652276	SPAG9	TRNA_Lys	SGOL2	ATF4	ANTXR2	DDR1	PTPRZ1
TMEM39B	MIR1915	CWC15	MIR17HG	ERVK13-1	NME1	ETV2	AOX1	HV452684	PRDM8	GTF2H4	GPR37
CCDC28B	MLLT10	ENDOD1	GPC5	KCTD5	UTP18	MLL4	BZW1	MIR1281	RASGEF1B	MICA	ZNF800
MARCKSL1	DNAJC1	AK055250	GPC6	PRSS27	HLF	ARHGAP33	FZD7	TOB2	HNRNPD	PRRC2A	ARF5
BSDC1	COMMD3	FAM76B	STK24	TCEB2	PCTP	LRFN3	BMPR2	POLR3H	ENOPH1	C6orf47	SND1
KIAA1522	BMI1	CCDC82	DOCK9	FLYWCH1	NOG	BC071809	NBEAL1	CSDC2	LIN54	LY6G6E	LRRC4
YARS	MSRB2	C11orf70	TM9SF2	KREMEN2	DGKE	ZFP30	CYP20A1	PMM1	COPS4	CLIC1	PRRT4
TRIM62	OTUD1	YAP1	ZIC5	PAQR4	COIL	YIF1B	ABI2	PPPDE2	COQ2	VARS	SMO
ZNF362	ARHGAP21	TMEM123	ZIC2	PKMYT1	MRPS23	GGN	RAPH1	SREBF2	NKX6-1	ZBTB12	AHCYL2
ZSCAN20	PRTFDC1	DYNC2H1	PCCA	TNFRSF12A	VEZF1	ACTN4	NRP2	TNFRSF13C	CDS1	PBX2	KLHDC10
ZMYM6	GAD2	AASDHPPT	TMTC4	ZSCAN10	AK126318	LOC643669	NDUFS1	NAGA	PTPN13	BRD2	CEP41
ZMYM4	ABI1	ELMOD1	ABHD13	ZNF213	DYNLL2	FBXO17	ZDBF2	SERHL2	LOC100506746	RING1	AK054623
NCDN	MASTL	RAB39A	IRS2	TRNA_Pseudo	MIR4736	PAK4	ADAM23	ARFGAP3	KLHL8	B3GALT4	PODXL
EIF2C4	MPP7	RDX	COL4A1	ZNF434	SUPT4H1	SAMD4B	MIR2355	TTLL12	HSD17B11	RGL2	SLC35B4
EIF2C3	LOC100507605	FDX1	RAB20	CREBBP	MTMR4	ZFP36	CREB1	SAMM50	PKD2	PHF1	AKR1B1
TEKT2	ZNF438	SIK2	CARS2	LOC100507501	PPM1E	MIR4530	METTL21A	LDOC1L	FAM13A	ZBTB9	C7orf49
ADPRHL2	ARHGAP12	TTC12	LINC00346	TFAP4	YPEL2	TRNA_Ile	CCNYL1	ARHGAP8	GPRIN3	HMGA1	WDR91
TRAPPC3	ITGB1	USP28	SOX1	GLIS2	DHX40	EID2	FZD5	PHF21B	PDLIM5	C6orf1	C7orf73
MAP7D1	LOC100505583	ZBTB16	AK055145	C16orf5	CLTC	PSMC4	C2orf80	FBLN1	RAP1GDS1	C6orf106	TRIM24
THRAP3	CREM	NNMT	ATP11A	AK055785	APPBP2	MIR641	IDH1	LOC554174	TSPAN5	SNRPC	KIAA1549
STK40	ZNF33A	RBM7	AX748036	NUDT16L1	PPM1D	SERTAD3	PIKFYVE	PPARA	METAP1	SCUBE3	TTC26
LSM10	CSGALNACT2	CADM1	PROZ	ZNF500	TBX2	NUMBL	MAP2	CELSR1	DNAJB14	ZNF76	UBN2
MRPS15	RASGEF1A	BUD13	PCID2	FAM86A	BRIP1	HNRNPUL1	UNC80	CERK	FLJ20021	PPARD	TBXAS1
MANEAL	ZNF487P	APOA1	LAMP1	CARHSP1	MRC2	TGFB1	BARD1	LOC90834	SLC39A8	TEAD3	LOC100134229
SF3A3	ALOX5	RNF214	GRTP1	C16orf72	DCAF7	BCKDHA	ATIC	ALG12	CENPE	LOC285847	DENND2A
FHL3		MGC13053	TMCO3	EMP2	MAP3K3	AK024119	IGFBP2	CRELD2	AK299501	SRPK1	BRAF
POU3F1	ZFAND4	MLL	RASA3	NUBP1	STRADA	GSK3A	PNKD	PIM3	HADH	KCTD20	MRPS33
MIR3659	ZNF488	TMEM25	UPF3A	CLEC16A	CCDC47	ERF	USP37	MAPK11	LEF1	CPNE5	AGK
AKIRIN1	MAPK8	ARCN1	CHAMP1	SNN	SMARCD2	CIC	WNT6	PLXNB2	LOC285456	AK096023	KIAA1147
MACF1	C10orf53	PHLDB1	ARHGEF40	TXNDC11	ERN1	MEGF8	WNT10A	FAM116B	PLA2G12A	MTCH1	SSBP1
KIAA0754	LOC100506939	HINFP	SUPT16H	MIR193B	TEX2	CNFN	DNAJB2	PPP6R2	ELOVL6	PIM1	FAM115A
HEYL	TFAM	CBL	TOX4	BFAR	DDX5	SRRM5	DES	LMF2	AP1AR	RNF8	CNTNAP2
CAP1	BICC1	MCAM	SALL2	NTAN1	SMURF2	PLAUR	STK11IP	NCAPH2	NEUROG2	FTSJD2	EZH2
RLF	CCDC6	PVRL1	DAD1	MIR3180-4	LRRC37A3	ZNF234	LOC440934	SHANK3	CAMK2D	MIR4462	PDIA4
ZMPSTE24	C10orf107	OAF	AJUBA	SYT17	AMZ2P1	PVRL2	FARSB	CRBN	UGT8	ZFAND3	ZNF786
ZNF643	RTKN2	ARHGEF12	CDH24	IQCK	CEP112	RELB	ACSL3	ITPR1	SNHG8	KCNK5	ZNF398
ZNF642	SIRT1	GRIK4	C14orf119	THUMPD1	PRKCA	MARK4	WDFY1	ARL8B	PRSS12	C6orf130	KRBA1
SCMH1	HERC4	UBASH3B	PPP1R3E	LOC81691	CACNG4	RTN2	SERPINE2	EDEM1	BC070391	NFYA	LOC285972
PPIH	TET1	HSPA8	BCL2L2	LYRM1	PSMD12	VASP	CUL3	SETD5	PRDM5	FOXP4	KCNH2
CLDN19	DDX50	SCN3B	ZFHX2	METTL9	NOL11	MIR642B	IRS1	BRPF1	NDNF	MDFI	NOS3
SLC2A1	VPS26A	NRGN	FAM158A	POLR3E	BPTF	AX746967	AK056332	OGG1	ANXA5	FRS3	SLC4A2
ELOVL1	NEUROG3	PKNOX2	PSME2	USP31	TRNA_Arg	SIX5	TRIP12	MKRN2	FGF2	PRICKLE4	SMARCD3
SZT2	SAR1A	DDX25	IPO4	UBFD1	AMZ2	FOXA3	SNORD82	RAF1	ANKRD50	MED20	GALNT11
ST3GAL3	PPA1	KCNJ1	NOVA1	PRKCB	PRKAR1A	IRF2BP1	BC069004	DQ587809	HSPA4L	CCND3	MLL3
DMAP1	LRRC20	APLP2	FOXG1	CACNG3	SSTR2	MYPOP	PTMA	NUP210	LARP1B	TRERF1	FABP5P3
PLK3	KIAA1274	ADAMTS15	SCFD1	RBBP6	COG1	NOVA2	GIGYF2	HDAC11	PGRMC2	UBR2	LOC100128822
TCTEX1D4	ADAMTS14	THYN1	HECTD1	LCMT1	C17orf80	CCDC8	DGKD	LOC285375	PCDH18	TBCC	PAXIP1
PTCH2	UNC5B	LOC283177	HEATR5A	ZKSCAN2	RPL38	MIR3191	ARL4C	SLC6A6	CCRN4L	MRPL2	INSIG1
ZSWIM5	SLC29A3	B4GALNT3	ARHGAP5	IL4R	MGC16275	PRR24	SH3BP4	ZFYVE20	RAB33B	PTK7	EN2
LOC400752	C10orf54	BC039168	AKAP6	XPO6	TTYH2	MEIS3	AGAP1	COL6A4P1	SCOC	SRF	RBM33
AKR1A1	DDIT4	LRTM2	NPAS3	SBK1	GPRC5C	SLC8A2	GBX2	DPH3	LOC100129858	ZNF318	RNF32
GPBP1L1	MICU1	DCP1B	SPTSSA	MAZ	NAT9	NAPA	UBE2F	KCNH8	ZNF330	TJAP1	LMBR1
RAD54L	P4HA1	TEAD4	SEC23A	PRRT2	USH1G	SEPW1	ESPNL	KAT2B	MMAA	LRRC73	NCAPG2
BEND5	ANXA7	BC047090	TRAPPC6B	KCTD13	C17orf28	LIG1	HES6	UBE2E2	ZNF827	POLH	ESYT2
FAF1	CAMK2G	DQ597527	FBXO33	TBX6	ICT1	C19orf68	ASB1	UBE2E1	ARHGAP10	VEGFA	ZNF596
CDKN2C	ADK	CCND2	LRFN5	AK097453	ATP5H	CCDC114	GPC1	MIR4442	NR3C2	HSP90AB1	ERICH1
RNF11	KAT6B	KCNA1	BC038722	CD2BP2	SLC16A5	SYNGR4	DUSP28	TGFBR2	DCLK2	TMEM151B	MIR596
EPS15	SAMD8	MRPL51	KLHDC2	TBC1D10B	ARMC7	PLEKHA4	RNPEPL1	STT3B	RPS3A	AARS2	ARHGEF10
ZFYVE9	ZNF503-AS1	NCAPD2	L2HGDH	ZNF764	CASKIN2	PPP1R15A	FARP2	CMTM7	FAM160A1	ENPP5	FLJ10661
ZCCHC11	RPS24	ZNF384	MAP4K5	PRR14	TSEN54	BAX	THAP4	CMTM6	TRIM2	MUT	MFHAS1
LRP8	ZMIZ1	COPS7A	TRIM9	FBRS	GALK1	NTF4	LOC728323	DYNC1LI1	MND1	PAQR8	TNKS
HSPB11	ZCCHC24	GPR162	GNG2	SRCAP	CDK3	DKKL1	ZCCHC3	TRIM71	KIAA0922	EFHC1	NEIL2
LRRC42	TSPAN14	RRPL13L	PTGER2	MIR4519	MXRA7	RPS11	SOX12	UBP1	SFRP2	GSTA5	CTSB

TMEM59
SSBP3
PARS2
PRKAA2
JUN
HSD52
BC030753
USP1
ATG4C
ROR1
CACHD1
BC035370
AK4
DNAJC6
LEPROT
IL12RB2
GADD45A
WLS
NEGR1
LRRIO3
ACADM
AK5
NEXN
BC037304
SSX2IP
SYDE2
HS2ST1
LMO4
GTF2B
CCBL2
LRRC8B
AK022898
RPL5
MTF2
LOC100131564
FNBP1L
MIR760
GCLM
ABCD3
F3
CNN3
ALG14
Mir 448
RWDD3
PTBP2
DPYD
SASS6
COL11A1
STXBP3
TMEM167B
SCARNA2
SYPL2
ATXN7L2
CYB561D1
AMPD2
AHCYL1
KCNC4
KCNA3
DDX20
RHOC
PPM1J
FAM19A3
SLC16A1
PHTF1
RSBN1
DCLRE1B
OLFML3
TRIM33
CSDE1
ATP1A1
ATP1A1OS
CD58
IGSF3
TRIM45
MAN1A2
FAM46C
SPAG17
SEC22B
NOTCH2NL
PIAS3
PRKAB2
CHD1L
BCL9
OTUD7B
VPS45
C1orf51
ADAMTSL4
MCL1
FAM63A
MLLT11
SCNM1
CGN
SNX27
MRPL9
S100A2
SNAPIN
SLC39A1
JTB
RAB13
UBAP2L
UBE2O1
ADAR
PMVK
SHC1
ZBTB7B
ADAM15
SLC50A1
MIR92B
MTX1
GBA
GON4L
UBOLN4
LMNA
MEF2D
BC005081
NES
CRABP2
RRNAD1
PEAR1
PIGM
NHLH1
DEDD
ATF6
OLFML2B
NOS1AP
UAP1
RGS5
PBX1
POGK
TADA1
RBSG4
POU2F1
SFT2D2
ATP1B1
METTL11B
DNM3
PRDX6
RABGAP1L
LOC100302401
ANGPTL1
MPP1
DNAJB5
STOML2
FAM214B
RUSC2
TESK1
CD72
RMRP
TPM2
TLN1
NPR2
ZCCHC7
FRMPD1
RG9MTD3
AQP7P1
AK096834
APBA1
KLF9
TMEM2
ZFAND5

FAM190B
AK097624
BMPR1A
PTEN
TNKS2
FUNDC2
FRA10AC1
PLCE1
TM9SF3
PIK3AP1
LCOR
FRAT1
FRAT2
ZDHHC16
MMS19
AVPI1
MARVELD1
ZFYVE27
GOLGA7B
ENTPD7
COX15
ERLIN1
SCD
LINC00263
FAM178A
NPM3
C10orf76
CUEDC2
MIR146B
TRIM8
ARL3
USMG5
NEURL
SFR1
SORCS3
SORCS1
DUSP5
RBM20
GPAM
TCF7L2
CASP7
FAM160B1
ATRNL1
PDZD8
FAM204A
C10orf46
NANOS1
PPAPDC1A
WDR11
FGFR2
NSMCE4A
ACADSB
METTL10
FLJ37035
FAM196A
PTPRE
BNIP3
DPYSL4
LRRC27
PWWP2B
C10orf91
INPP5A
C10orf93
KNDC1
MIR3944
PAOX
RIC8A
SIRT3
ATHL1
B4GALNT4
RNH1
HRAS
MIR210
LOC143666
SLC25A22
PIDD
RPLP2
PNPLA2
CD151
TSPAN4
AK126380
CD81
KCNQ1
KCNQ1DN
CDKN1C
PHLDA2
OSBPL5
ART5
RHOG
CNGA4
SMPD1
ILK
DCHS1
NLRP14
TUB
TMEM41B
LOC644656
DKK3
TEAD1
FAR1
PDE3B
PLEKHA7
PIK3C2A
NUCB2
KCNJ11
ABCC8
SAAL1
GTF2H1
TMEM86A
NAV2
NELL1
CCDC34
C11orf46
MPPED2
OSER1
CSTF3
HIPK3
CD59
CAPRIN1
PDHX
SLC1A2
FJX1
TRIM44
API5
MIR670
ACCS
CD82
TP53I11
MAPK8IP1
PHF21A
DGKZ
MDK
ARHGAP1
ZNF408
LRP4
ARFGAP2
DDB2
PSMC3
CELF1
MTCH2
PTPRJ
ISCA1
ZCCHC6
GAS1
DAPK1
NXNL2
MIR4289
SECISBP2
GADD45G
AUH
IPPK
C9orf89
NINJ1
PHF2
DL491118
MIR23B
MIR3074
C9orf102
LINC00092
ZNF367

SLC2A3
FAM86FP
PHC1
A2M
GABARAPL1
LRP6
DUSP16
CREBL2
DDX47
GPRC5A
EPS8
PLEKHA5
AEBP2
PDE3A
KCNJ8
CMAS
KIAA0528
RASSF8
BHLHE41
STK38L
KLHDC5
CAPRIN2
LOC100287314
TSPAN11
DENND5B
AMN1
C12orf35
BICD1
ALG10B
CPNE8
KIF21A
LRRK2
GXYLT1
YAF2
PRICKLE1
TMEM117
ANO6
ARID2
SCAF11
HDAC7
COL2A1
CCNT1
CACNB3
ARF3
PRKAG1
TUBA1A
C1QL4
SPATS2
KCNH3
PRPF40B
FMNL3
BCDIN3D
LOC283332
AQP5
ATF1
SLC11A2
CSRNP2
TFCP2
SCN8A
ACVR1B
C12orf44
EIF4B
PCBP2
LOC100240735
METTL7B
CD63
GDF11
AX747140
ESYT1
SMARCC2
ANKRD52
SPRYD4
BAZ2A
PTGES3
NACA
PRIM1
ZBTB39
NAB2
Mir 616
CTDSP2
USP15
XPOT
RASSF3
GNS
TMBIM4
CAND1
DYRK2
RAP1B
CPSF6
RAB3IP
CNOT2
KCNMB4
PHLDA1
NAP1L1
OSBPL8
ZDHHC17
E2F7
DUSP6
POC1B-GALNT
LOC338758
BTG1
EEA1
NUDT4
AK130911
TMCC3
FGD6
METAP2
ELK3
SLC9A7P1
LOC643770
UHRF1BP1L
ACTR6
DEPDC4
ARL1
DRAM1
C12orf42
NT5DC3
C12orf73
HCFC2
NFYB
CHST11
KIAA1033
C12orf75
NUAK1
CKAP4
TCP11L2
C12orf23
CMKLR1
SELPLG
CORO1C
SSH1
SVOP
ALKBH2
C12orf34
GLTP
ATP2A2
PPTC7
PPP1CC
FAM109A
SH2B3
ALDH2
ARPC5L
PBX3
FAM125B
LMX1B
ZBTB34
ANGPTL2
FAM129B
STXBP1
MIR3960
FAM102A
LOC100289019
PTGES2
C9orf16
CERCAM
SPTAN1
ZDHHC12
ZER1
TBC1D13

GPR137C
ERO1L
STYX
GNPNAT1
FERMT2
DDHD1
GMFB
CGRRF1
SAMD4A
SOCS4
MAPK1IP1L
PELI2
DAAM1
GPR135
C14orf135
HIF1A
SGPP1
ESR2
ZBTB1
PLEKHG3
SPTB
GPHN
MPP5
PIGH
ACTN1
GALNTL1
KIAA0247
SRSF5
MED6
PCNX
DCAF4
RBM25
ACOT6
PNMA1
ISCA2
LTBP2
PGF
FOS
LOC731223
JDP2
FLVCR2
ANGEL1
IRF2BPL
KIAA1737
GSTZ1
ISM2
PTPN21
ZC3H14
KCNK13
CALM1
C14orf159
SMEK1
FBLN5
ATXN3
LGMN
GOLGA5
MOAP1
OTUB2
PPP4R4
DICER1-AS1
CLMN
C14orf132
C14orf129
AK7
BCL11B
SETD3
CCDC85C
CYP46A1
EVL
YY1
WDR25
BC148240
DIO3
PPP2R5C
DYNC1H1
TRNA Ile
ANKRD9
RCOR1
TRAF3
CDC42BPB
EIF5
MARK3
CKB
BAG5
PPP1R13B
ASPG
MIR4710
INF2
ADSSL1
SIVA1
AKT1
ZBTB42
KIAA0284
CDCA4
NUDT14
MTA1
NIPA2
WHAMMP3
UBE3A
GABRG3
WHAMMP2
APBA2
BC043570
TJP1
DKFZP434L187
FAN1
KLF13
OTUD7A
CHRFAM7A
DKFZp434L187
RYR3
LPCAT4
LOC100507466
SPRED1
THBS1
GPR176
ANKRD63
AL832909
DISP2
CHST14
DLL4
INO80
CHP
OIP5
ITPKA
MAPKBP1
STARD9
TP53BP1
MAP1A
C15orf63
CTDSPL2
EIF3J
TRIM69
MYEF2
SECISBP2L
GALK2
USP8
AP4E1
MAPK6
MYO5A
ONECUT1
BRD3
WDR5
RNU6ATAC
RXRA
COL5A1
C9orf116
CAMSAP1
C9orf69
OSOX2
SDCCAG3
AGPAT2
FAM69B
SNORA17
TMEM141
C9orf172
FBXW5
TUBB4B
COBRA1
NRARP

STX4
ZNF668
BCKDK
FUS
GPT2
DNAJA2
LONP2
N4BP1
CBLN1
BRD7
NKD1
CYLD
SALL1
IRX3
CRNDE
IRX5
MMP2
LOC283856
NUP93
HERPUD1
RSPRY1
CX3CL1
DOK4
C16orf80
NDRG4
CDH8
BEAN1
CKLF
CBFB
B3GNT9
LRRC29
HSD11B2
CTCF
NUTF2
EDC4
PSKH1
SLC12A4
ESRP2
PLA2G15
PRMT7
ZFP90
CDH3
SNTB2
TERF2
MIR1538
PDPR
ST3GAL2
MTSS1L
FTSJD1
AP1G1
DHODH
PMFBP1
LOC283922
CLEC18B
WDR59
ZNRF1
TMEM231
ADAMTS18
CENPN
GCSH
GAN
CMIP
MLYCD
NECAB2
MBTPS1
HSDL1
TAF1C
KLHL36
LOC400548
MAP1LC3B
BANP
ZFPM1
ZC3H18
SNAI3
CTU2
APRT
C16orf7
FANCA
FAM57A
NXN
BHLHA9
YWHAE
MIR22
DPH1
MIR132
MIR212
HIC1
SRR
MNT
METTL16
PAFAH1B1
KIAA0664
TRPV3
C17orf85
CAMKK1
UBE2G1
PELP1
ARRB2
PLD2
ENO3
ZNF232
LOC728392
FAM64A
TXNDC17
MED31
C17orf49
DLG4
DVL2
GABARAP
SLC2A4
NEURL4
KCTD11
C17orf61
POLR2A
MPDU1
SAT2
ATP1B2
EFNB3
DNAH2
KDM6B
AX746771
KCNAB3
TRNA Leu
HES7
PER1
VAMP2
LINC00324
CTC1
LOC100128288
MYH10
CCDC42
NTN1
TMEM220
MAP2K4
HS3ST3A1
HS3ST3B1
ZNF286A
PIGL
CENPV
C17orf76-AS1
FAM211A
SMS
PRDX4
POLA1
PRRG1
RPGR
USP9X
DDX3X
MAOB
KDM6A
BC015977
CHST7
SLC9A7
RP2
ELK1
TFE3
LOC158572
KDM5C
FAM120C
FAM123B

MIR636
FLJ45079
SOCS3
CYTH1
USP36
ENGASE
CBX2
CBX8
TBC1D16
CCDC40
EIF4A3
BAIAP2
MIR1250
AZI1
BAHCC1
DQ585569
C17orf70
TSPAN10
FAM195B
P4HB
PYCR1
NOTUM
ASPSCR1
GPS1
DUS1L
FASN
CCDC57
SECTM1
HEXDC
C17orf62
TRNA Met
WDR45L
RAB40B
FN3KRP
TBCD
COLEC12
C18orf56
YES1
SMCHD1
EMILIN2
LINC00526
LOC339290
LOC645355
TMEM200C
PTPRM
RAB12
CCDC165
NDUFV2
ANKRD12
TWSG1
APCDD1
GNAL
TUBB6
AFG3L2
SLMO1
PTPN2
SEH1L
FAM210A
MC5R
C18orf8
NPC1
ZNF521
CHST9
B4GALT6
TRAPPC8
FAM59A
C18orf34
ASXL3
MAPRE2
ZNF271
ZNF24
INO80C
GALNT1
C18orf21
SLC39A6
ELP2
FHOD3
TPGS2
SETBP1
C18orf25
PIAS2
SMAD7
MAPK4
SMAD4
MEX3C
C18orf54
TXNL1
ONECUT2
NEDD4L
LMAN1
PMAIP1
RNF152
ZCCHC2
PHLPP1
BCL2
KDSR
DOK6
SOCS6
NETO1
TIMM21
CNDP2
ZADH2
FLJ44881
BC070490
LOC100131655
ATP9B
NFATC1
TXNL4A
PARD6G
C2CD4C
TPGS1
HCN2
FGF22
RNF126
PALM
KISS1R
ARID3A
WDR18
CNN2
STK11
C19orf26
ATP5D
MIDN
RPS15
REEP6
MBD3
TCF3
KLF16
ADAT3
BTBD2
MKNK2
MOB3A
AP3D1
DOT1L
SF3A2
SPPL2B
GADD45B
SLC39A3
THOP1
TLE6
TLE2
AIFM1
ZNF280C
MST4
GPC4
MIR106A
CCDC160
HPRT1
LOC100506757
LINC00086
MMGT1
HTATSF1
MAMLD1
MTM1
CD99L2
VMA21
ZNF275
SLC6A8
PDZD4
TMEM187

RRAS
SCAF1
PRMT1
AP2A1
PNKP
AKT1S1
FLJ26850
POLD1
JOSD2
SHANK1
SNORD88C
MIR99B
ZNF480
ZNF610
ZNF808
ZNF816
ZNF160
PRKCG
CACNG6
PRPF31
MBOAT7
TSEN34
LENG8
CDC42EP5
PPP1R12C
SYT5
PPP6R1
BRSK1
TMEM190
ZNF628
FIZ1
ZNF581
CCDC106
ZNF542
ZNF805
BC063675
ZNF446
FAM110C
ACP1
TSSC1
RNASEH1
SOX11
LINC00487
ID2
MBOAT2
ITGB1BP1
ADAM17
YWHAQ
GRHL1
KLF11
CYS1
RRM2
HPCAL1
SNORA80B
KCNF1
POLC3
ROCK2
E2F6
TRIB2
PUM2
GDF7
KLHL29
PFN4
ADCY3
DNAJC27
EFR3B
DTNB
ASXL2
KIF3C
FAM59B
HADHB
KCNK3
C2orf18
DPYSL5
MAPRE3
OST4
KHK
EIF2B4
ZNF513
FNDC4
AK124439
MRPL33
LOC100302650
BRE
FOSL2
WDR43
YPEL5
LBH
SPAST
SLC30A6
LOC100288911
STRN
CDC42EP3
ATL2
HNRPLL
SRSF7
SOS1
PKDCC
EML4
TRNA Ile
LOC100129726
LRPPRC
PPM1B
EPAS1
SOCS5
MSH2
MSH6
FBXO11
FOXN2
PPP1R21
ASB3
PSME4
SPTBN1
EML6
RTN4
CCDC88A
FLJ16341
KIAA1841
XPO1
CCT4
VPS54
LGALSL
AFTPH
SERTAD2
AK097952
RAB1A
SPRED2
FLJ16124
C1D
PPP3R1
GFPT1
AAK1
MXD1
PCBP1
LOC100133985
FAM136A
ADD2
VAX2
AK125051
MOGS
HTRA2
RAD23B
KLF4
IKBKAP
UGCG
PTBP3
C9orf80
SNX30
FKBP15
WDR31
ALAD
C9orf43
ZNF618
COL27A1
ATP6V1G1
PHF19
TRAF1
LHX6
ZBTB26
DENND1A

RBCK1
CSNK2A1
RAD21L1
NSFL1C
NOP56
EBF4
MRPS26
ProSAPiP1
C20orf194
ATRN
HSPA12B
C20orf27
CDC25B
RNF24
TRMT6
CRLS1
TMX4
PLCB4
SNAP25
JAG1
LOC339593
KIF16B
DSTN
C20orf72
PET117
DTD1
SLC24A3
NAA20
INSM1
RALGAPA2
PLK1S1
NKX2-2
NXT1
GZF1
ABHD12
NINL
BCL2L1
POFUT1
KIF3B
ASXL1
C20orf112
LOC284804
DNMT3B
SNTA1
CBFA2T2
CHMP4B
RALY
GGT7
CEP250
RBM39
DLGAP4
TGIF2
KIAA0889
SRC
CTNNBL1
TTI1
SNHG11
SLC32A1
DHX35
MAFB
BC035080
ZHX3
LPIN3
TOX2
LOC100505783
YWHAB
PABPC1L
STK4
SLPI
SDC4
SNX21
ZNF335
SLC12A5
NCOA5
TP53RK
SLC2A10
EYA2
NCOA3
PREX1
ARFGEF2
B4GALT5
SLC9A8
RNF114
SNAI1
CEBPB
PARD6B
BCAS4
ADNP
AK056432
ZNF217
PFDN4
DOK5
FAM210B
C20orf43
MIR4325
RBM38
RAB22A
STX16
TAF4
LSM14B
PSMA7
HRH3
OSBPL2
ADRM1
GATA5
SLCO4A1
C20orf20
COL9A3
C20orf11
BHLHE23
MIR124-3
YTHDF1
BIRC7
PPDPF
PRIC285
GMEB2
STMN3
ARFRP1
SLC2A4RG
ABHD16B
DNAJC5
UCKL1
ZNF512B
SOX18
NRIP1
USP25
CXADR
C21orf91
NCAM2
MRPL39
JAM2
ADAMTS1
SCAF4
C21orf59
SYNJ1
GCFC1
OLIG2
IFNAR2
LOC100288432
IFNGR2
TMEM50B
NCRNA00269
IGBP1
CITED1
JPX
SLC16A2
KLHL4
PCDH19
XKRX
BEX4
PRPS1
IRS4
KCNE1L
ACSL4
Mir 633
ALG13
WDR44
DOCK11
IL13RA1
ZCCHC12

PLCD1
TTC21A
MYRIP
FLJ33065
NKTR
KBTBD5
KIF15
TMEM158
LARS2
LIMD1
LZTFL1
TMIE
PTPN23
SCAP
CDC25A
NME6
SHISA5
P4HTM
ORICH1
LAMB2
CCDC71
USP4
BSN-AS2
IP6K1
HYAL2
CISH
VPRBP
GRM2
PCBP4
ABHD14B
DUSP7
POC1A
GLYCTK
DNAH1
BAP1
NISCH
PRKCD
TKT
CACNA1D
ACTR8
LRTM1
SPATA12
APPL1
ABHD6
PDHB
KCTD6
PTPRG
PRICKLE2
LRIG1
ARL6IP5
RYBP
GXYLT2
FAM86DP
GBE1
PROS1
AK296966
CLDND1
CPOX
TOMM70A
KIAA1524
DPPA4
PVRL3
PLCXD2
TAGLN3
C3orf17
BOC
KIAA2018
AX747798
B4GALT4
POGLUT1
C3orf15
STXBP5L
FAM162A
KPNA1
PTPLB
MYLK
CCDC14
KALRN
HEG1
ZNF148
SNX4
OSBPL11
LOC100125556
TPRA1
MGLL
RUVBL1
RPN1
RAB7A
ACAD9
CCDC48
CNBP
C3orf37
IFT122
PLXND1
LOC100507032
FAM86HP
RAB6B
EPHB1
PPP2R3A
MSL2
STAG1
SLC25A36
SPSB4
ACPL2
RASA2
ATP1B3
ATR
PLS1
TRPC1
AK093381
U2SURP
C3orf58
PLSCR1
HLTF
PFN2
TSC22D2
SIAH2
RAP2B
SSR3
TIPARP-AS1
LOC730091
LOC339894
C3orf80
LOC646168
GOLIM4
LOC100128164
PRKCI
SKIL
FNDC3B
NLGN1
U4
TBL1XR1
BC032034
ZNF639
ACTL6A
USP13
SOX2
ATP11B
DCUN1D1
YEATS2
MAP6D1
ENTPD8
WDR85
ZMYND19
ARRDC1
PRKX
TBL1X
SHROOM2
WWC3
MSL3
FRMPD4
RAB9A
GEMIN8
MOSPD2
RBBP7
REPS2
NHS
PHKA2
GPR64
PDHA1

GUCY1B3
PDGFC
TMEM144
NAF1
MSMO1
CPE
SH3RF1
CLCN3
MFAP3L
SAP30
VEGFC
MGC45800
DCTD
C4orf38
CDKN2AIP
LOC389247
ING2
STOX2
IRF2
CASP3
MLF1IP
SLC25A4
KIAA1430
SNX25
PDLIM3
UNQ2560
MTNR1A
FAT1
FRG1
PDCD6
CEP72
TPPP
NKD2
SLC6A19
SLC6A3
IRX2
IRX1
ADAMTS16
KIAA0947
SRD5A1
PAPD7
ADCY2
LOC100505806
MTCP1NB
ANKRD33B
DAP
MYO10
BASP1
CDH10
LOC643401
CDH6
PDZD2
GOLPH3
ZFR
U6
RAI14
DNAJC21
NADKD1
RICTOR
TTC33
C5orf28
FST
SNX18
IDAS
MAP3K1
MIER3
GPBP1
PLK2
ZSWIM6
BC039381
KIF2A
ERBB2IP
PIK3R1
MRPS36
TAF9
MAP1B
RGNEF
ENC1
WDR41
AP3B1
LHFPL2
HOMER1
AX746944
THBS4
SSBP2
ATG10
VCAN
RASA1
MBLAC2
LYSMD3
GPR98
AK091866
NR2F1
RHOBTB3
LNPEP
RGMB
LOC100289230
SLCO4C1
C5orf30
EFNA5
MAN2A1
CAMK4
KCNN2
FEM1C
TMED7-TICAM
CDO1
AP3S1
SEMA6A
DMXL1
HSD17B4
SRFBP1
SNX2
ZNF608
ALDH7A1
LMNB1
PRRC1
FLJ33630
CDC42SE2
FNIP1
RAD50
IL13
SHROOM1
AFF4
HSPA4
VDAC1
TCF7
UBE2B
PCBD2
NEUROG1
HNRNPA0
GFRA3
REEP2
EGR1
CTNNA1
MATR3
CXXC5
NRG2
BC042448
RELL2
KIAA0141
SPRY4
DOLK
SH3GLB2
AX747401
LOC100506190
ASB6
PTGES
GPR107
NCS1
ASS1
LOC100272217
PRDM12
C9orf9
SURF6
SURF1
SURF4
C9orf7
DBH
VAV2
LINC00094

GCLC
LRRC1
BAI3
LMBRD1
RIMS1
DQ590492
TMEM30A
SENP6
PHIP
C6orf7
ELOVL4
FAM46A
IBTK
UBE2CBP
DOPEY1
ME1
SYNCRIP
ZNF292
AKIRIN2
PNRC1
UBE2J1
BACH2
EPHA7
MANEA
UFL1
KLHL32
POU3F2
FBXL4
C6orf168
USP45
PREP
C6orf203
BEND3
PDSS2
SOBP
SEC63
AF520419
FOXO3
CD164
PPIL6
ZBTB24
WASF1
SLC16A10
FYN
MARCKS
RWDD1
ZUFSP
NKAIN2
CENPW
C6orf174
PTPRK
TMEM200A
EPB41L2
MED23
ENPP1
MOXD1
RPS12
AHI1
IFNGR1
AK124173
HEBP2
CITED2
VTA1
GPR126
HIVEP2
UTRN
FBXO30
C6orf103
SASH1
UST
PPIL4
PCMT1
RAET1K
ULBP3
C6orf211
RGS17
SCAF8
U8
TMEM242
SNX9
SYNJ2
DYNLT1
FNDC1
SOD2
WTAP
TCP1
IGF2R
MAP3K4
CAHM
OKI
C6orf118
PDE10A
SFT2D1
BRP44L
RNASET2
FGFR1OP
AL832737
WDR27
PHF10
DLL1
GET4
INTS1
MAFK
FW339998
SNX8
EIF3B
BRAT1
TTYH3
SDK1
FOXK1
WIPI2
BC140723
USP42
RAC1
KDELR2
ZNF853
NDUFA4
PHF14
ARL4A
ANKMY2
TSPAN13
ITGB8
SP4
CDCA7L
C7orf30
TRA2A
MPP6
MIR148A
CBX3
LOC441204
JAZF1
WIPF3
PLEKHA8
ZNRF2
NOD1
GGCT
KBTBD2
EEPD1
AMPH
LINC00265
CDK13
C9orf41
PCSK5
GNAQ
TLE4
BC036431
FRMD3
GKAP1
RMI1
AGTPBP1
LOC389765
NAA35
ANP32B
ANKS6
NR4A3
TEX10
C9orf30
ZNF189
RNF20
SLC44A1

TUSC3
EFHA2
ZDHHC2
VPS37A
SLC7A2
MTUS1
PCM1
PSD3
INTS10
ATP6V1B2
C8orf58
EGR3
ENTPD4
DOCK5
KCTD9
PNMA2
PTK2B
CLU
SCARA3
FBXO16
BC043205
HMBOX1
DUSP4
MIR3148
GSR
WRN
ZNF703
AX747135
GPR124
WHSC1L1
FGFR1
PLEKHA2
GOLGA7
KAT6A
PLAT
THAP1
HOOK3
HGSNAT
KIAA0146
MCM4
TCEA1
MRPL15
TGS1
DL492700
CHCHD7
IMPAD1
SDCBP
GGH
AK093370
LOC100130155
CYP7B1
MYBL1
LOC645895
SNORD87
ARFGEF1
TRAM1
KCNB2
RDH10
STAU2
UBE2W
TMEM70
PEX2
FAM164A
HEY1
ZBTB10
PAG1
E2F5
CA2
FAM82B
MMP16
TMEM55A
FAM92A1
PDP1
GEM
KIAA1429
DPY19L4
TP53INP1
AX747981
PLEKHF2
MTDH
RNF19A
BC009500
PABPC1
ZNF706
RRM2B
UBR5
KLF10
AK127183
AZIN1
LRP12
OXR1
TTC35
TMEM74
WDR67
FBXO32
RNF139
SOLE
TRIB1
FAM84B
KCNQ3
ST3GAL1
KCNK9
EIF2C2
PTK2
MIR4472-1
ARC
PSCA
GLI4
GSDMD
TSTA3
MAPK15
PUF60
CYC1
SCRT1
ADCK5
KIFC2
FOXH1
ARHGAP39
ZNF250
C8orf33
GLDC
MPDZ
FREM1
SNAPC3
C9orf93
BNC2
MTAP
C9orf82
C9orf72
ACO1
NDUFB6
DNAJA1
B4GALT1
BAG1
NFX1
PTENP1
UBE2R2
KIAA1161
C9orf23
SIGMAR1
CCL27
EMD
GDI1
PLXNA3
UBL4A
DKC1
ZDHHC9
UTP14A
LOC729609
ZNF462
ANXA1
TDRD7
PHYHD1
EXD3
PJA1
FLNA
RPL35

Appendix Table S4.

Twenty five genes that were contained in the biological process 'Regulation of dendrite spine morphogenesis' and detected in both LDB2 and EGR1 ChIP analyses

Gene Symbol	Protein
CAPRIN1	Caprin-1
CAMK2B	Calcium/calmodulin-dependent protein kinase type II subunit beta
ARC	Activity-regulated cytoskeleton-associated protein
CAPRIN2	Caprin-2
SRCIN1	SRC kinase signaling inhibitor 1
SHANK3	SH3 and multiple ankyrin repeat domains protein 3
ARHGAP33	Rho GTPase-activating protein 33
OPA1	Dynamin-like 120 kDa protein, mitochondrial
PDLIM5	PDZ and LIM domain protein 5
DBN1	Drebrin
PTEN	Phosphatidylinositol 3,4,5-trisphosphate 3-phosphatase and dual-specificity protein phosphatase PTEN
PAFAH1B1	Platelet-activating factor acetylhydrolase IB subunit alpha
ITPKA	Inositol-trisphosphate 3-kinase A
CFL1	Cofilin-1
KALRN	Kalirin
LRRK2	Leucine-rich repeat serine/threonine-protein kinase 2
BAIAP2	Brain-specific angiogenesis inhibitor 1-associated protein 2
PPP1R9A	Neurabin-1
LRP8	Low-density lipoprotein receptor-related protein 8
CTTN	Src substrate cortactin
DNM3	Dynamin-3
NLGN1	Neuroligin-1
CDK5R1	Cyclin-dependent kinase 5 activator 1
ABI2	Abl interactor 2
UBE3A	Ubiquitin-protein ligase E3A

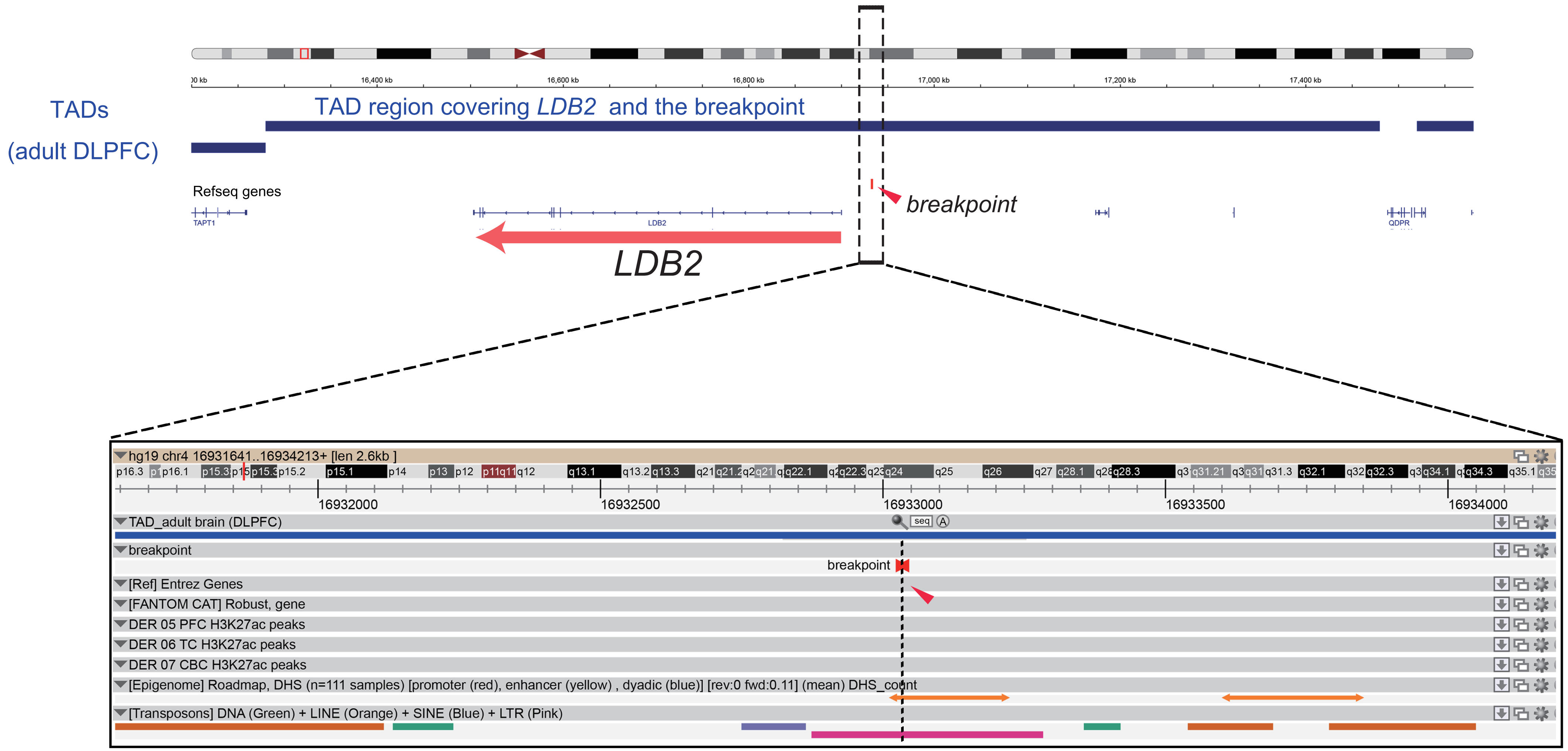

Appendix Figure S1: TAD map

TAD (topologically associating domain) structure around the chromosome 4 breakpoint of the proband is shown. The red arrowhead indicates the position of the breakpoint. Note that the breakpoint in chromosome 13 is within so-called ‘gene desert’.

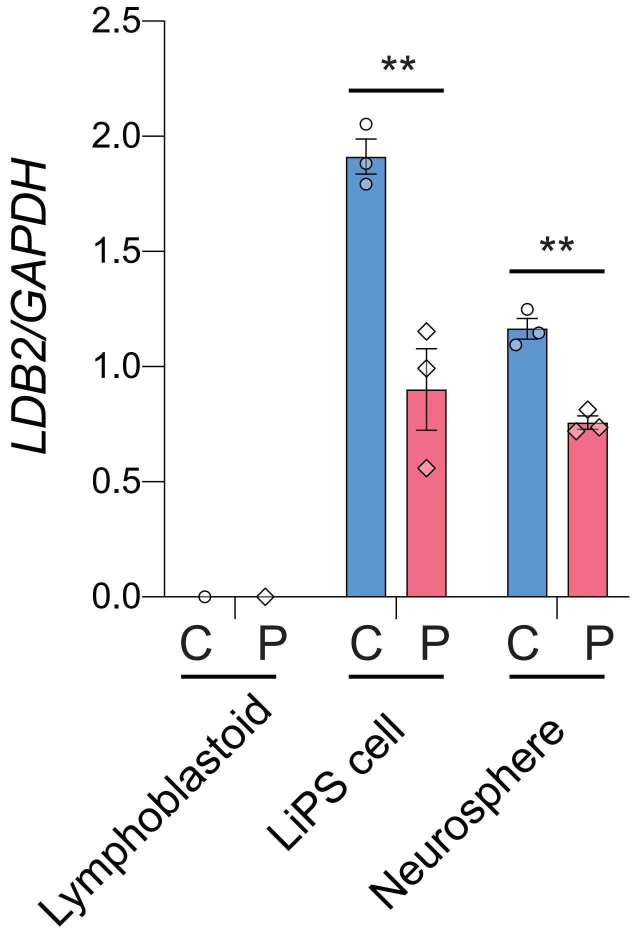

Appendix Figure S2: Reduced *LDB2* expression in the patient derived cells

Quantitative RT-PCR detected *LDB2* expression in the patient's lymphoblastoid cell-derived iPS (LiPS) cells and neurospheres differentiated from the LiPS cells. Three independent LiPS clones each from the patient (P) and a healthy control (C; 39 years old, male) were examined. **; $p < 0.01$ (t-test). Data are shown as means ± S.E.M (n = 3 each, lymphoblastoid and neurosphere). There are no biological replicates in lymphoblastoid cells.

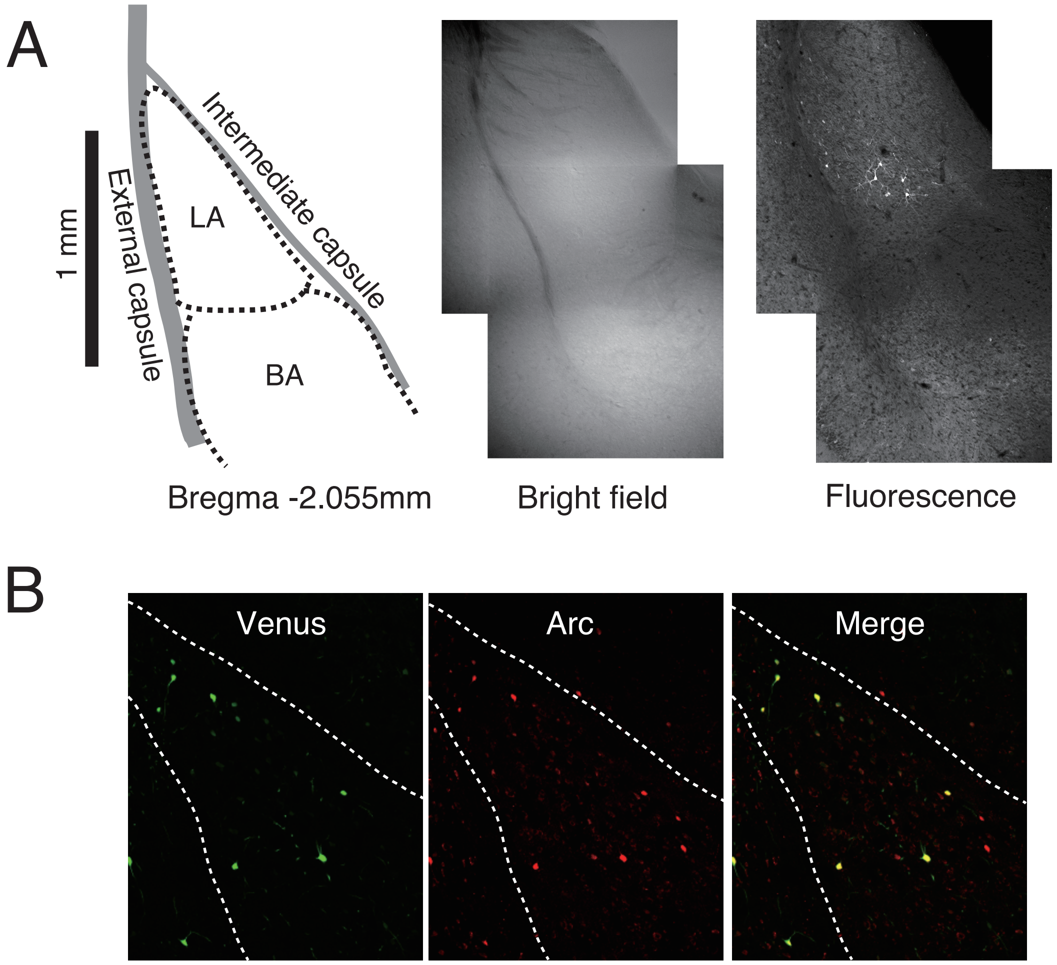

Appendix Figure S3: Analysis of neuronal activity in the Arc-Venus mouse

A. A schematic image of the basolateral complex of the amygdala (left), and bright-field (middle) and Venus-fluorescence (right) images in the corresponding area.

B. Venus fluorescence (green) counterstained immunohistochemically by an anti-Arc antibody (red) in the LA.

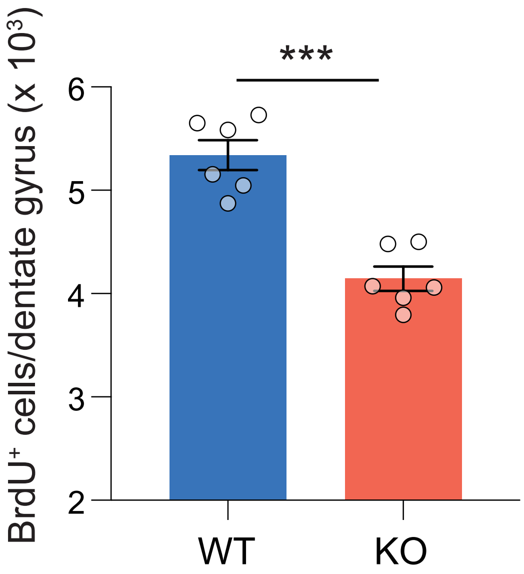

Appendix Figure S4: Abnormality of neurogenesis in the hippocampus of *Ldb2* KO animals

Neurogenesis in the hippocampal dentate gyrus was evaluated by the incorporation of BrdU. BrdU-positive cells from three animals for each genotype were separately counted. Data were shown as means with error bars representing SE. ***; $P < 0.001$, Student *t*-test (n = 6 for each genotype, 3 animals x both sides).
