# Supplementary material for: Cooperation of LIM domain‐binding 2 (LDB2) with EGR in the pathogenesis of schizophrenia
Source: EMBO Mol Med. 2021 Mar 3;13(4):e12574. doi: 10.15252/emmm.202012574 (PMC8033514; doi:10.15252/emmm.202012574)
Supplement: Supplementary file 2 — Expanded View Figures PDF [file EMMM-13-e12574-s002.pdf]

# Expanded View Figures

**Figure EV1. Basic Characterization of _Ldb2_ KO mice.**

A Genotyping of WT, heterozygous (HET), and homozygous (KO) mice by genomic PCR using DNA extracted from the tails.
B Quantitative RT–PCR did not detect the Ldb2 transcripts in the bran from KO. Note that no compensatory upregulation of the _Ldb1_ transcripts was seen in KO.
C Loss of the 48 and 35 kDa bands detected by the LDB2/Ldb2 polyclonal antibody in the two brain region from KO in Western blot analysis.
D No gross abnormalities were seen in KO.
E No apparent abnormalities were seen in the histological architecture of the brain from KO. Hematoxylin and eosin (HE) and Nissl staining were conducted using sagittal sections of the brains. Magnified image (Nissl staining) of the hippocampus is presented at bottom.
F Brain sections (the cerebral cortex, hippocampus, amygdala, olfactory bulb, striatum, thalamus, and cerebellum) form WT and KO mice were stained with anti-Ldb2 antibody.


© 2021 The Authors

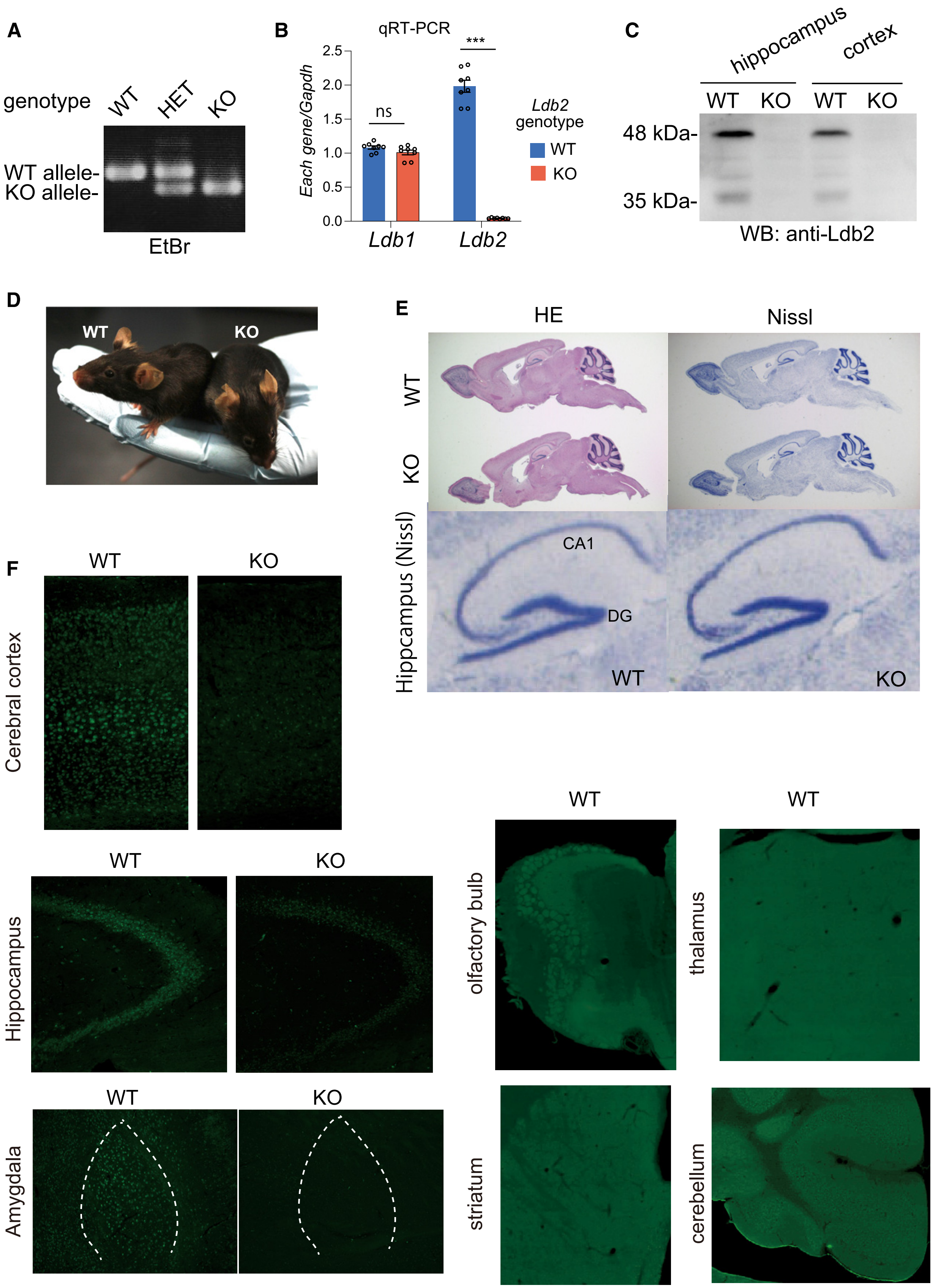
A
genotype
WT
HET
KO
WT allele-
KO allele-
EtBr
B
qRT-PCR
Each gene/Gapdh
2.5
2.0
1.5
1.0
0.5
0.0
ns
***
Ldb1
Ldb2
Ldb2 genotype
WT
KO
C
hippocampus
cortex
WT
KO
WT
KO
48 kDa-
35 kDa-
WB: anti-Ldb2
D
WT
KO
E
HE
Nissl
WT
KO
Hippcampus (Nissl)
CA1
DG
WT
KO
F
WT
KO
Cerebral cortex
WT
KO
Hippocampus
WT
KO
Amygdala
WT
WT
olfactory bulb
thalamus
striatum
cerebellum

**Figure EV1.**

**Figure EV2. mEPSCs recorded in LA neurons of Venus-positive or -negative cells in *Ldb2* KO mice.**

A Sample traces of mEPSCs from LA neurons in brain slices of AV-WT/ Venus$^{+}$, AV-WT/ Venus$^{-}$, AV-KO/ Venus$^{+}$ and AV-KO/ Venus$^{-}$ mice.

B Neither median amplitudes (upper panel) nor mean frequency (lower panel) of mEPSCs was statistically different between the genotypes.

C Scatter plots of Venus fluorescence intensities of patch-clamped cells. There was no significant difference in fluorescent intensities of Venus-positive cells between AV-KO mice and their AV-WT littermates (*t*-test, $n$ = 11/group).


© 2021 The Authors

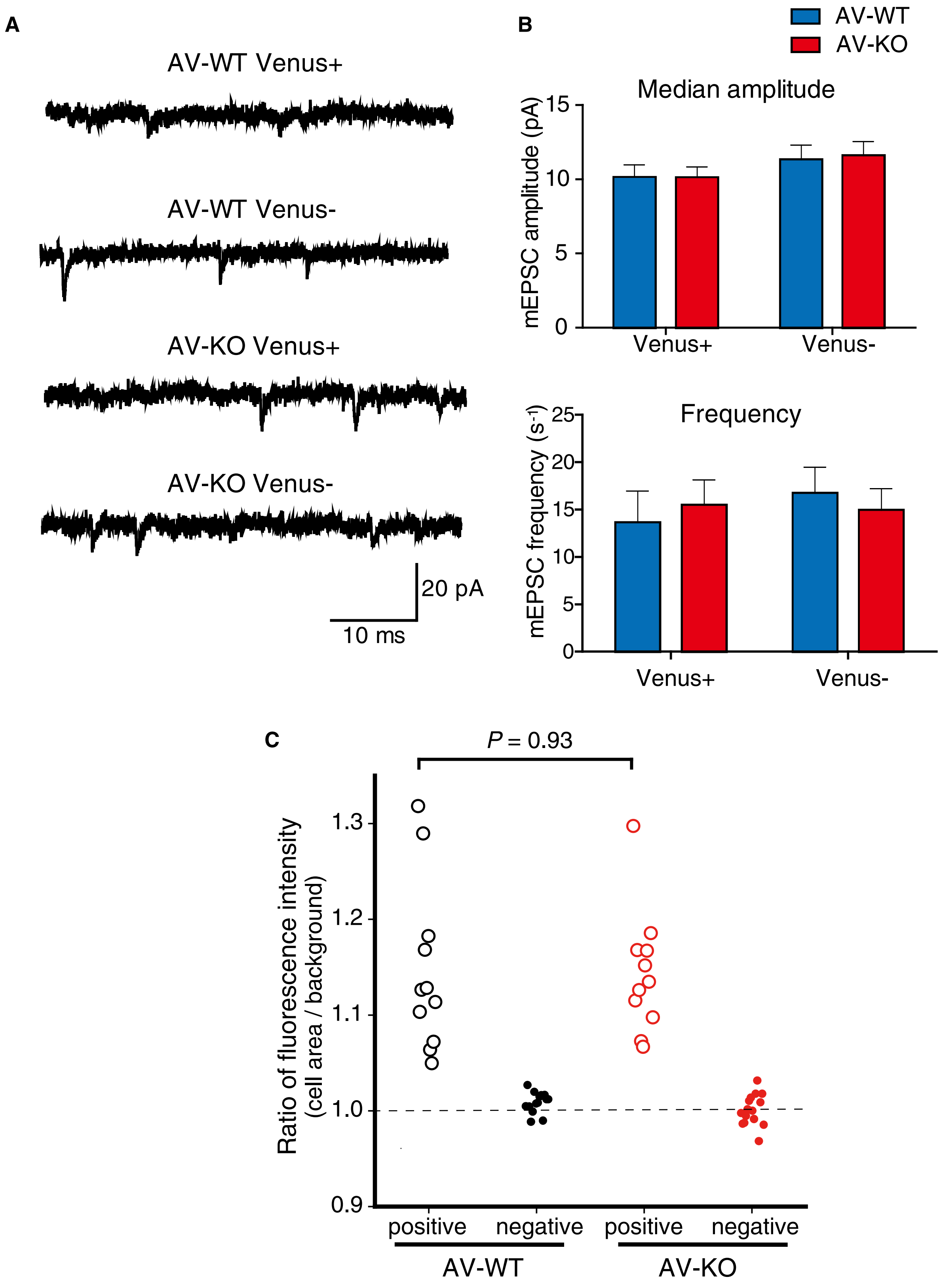
A
AV-WT Venus+
AV-WT Venus-
AV-KO Venus+
AV-KO Venus-
20 pA
10 ms
B
AV-WT
AV-KO
Median amplitude
mEPSC amplitude (pA)
15
10
5
0
Venus+
Venus-
Frequency
mEPSC frequency (s-1)
25
20
15
10
5
0
Venus+
Venus-
C
P = 0.93
Ratio of fluorescence intensity (cell area / background)
1.3
1.2
1.1
1.0
0.9
positive
negative
positive
negative
AV-WT
AV-KO

**Figure EV2.**

**Figure EV3. A schematic representation of behavioral experiments and Venus fluorescence counterstained by cell markers in the LA.**

A A schematic representation of the experimental procedure. Mice were moved from their home cage to the experimental room at least 3 h before conditioning. Mice of the Unpaired and Paired groups received conditioning (see the Methods section), while mice of the Naïve group were kept in their home cage in the experimental room. Mice were then transcardially perfused with 4% PFA 3 h after the fear conditioning.

B A typical Venus fluorescence image of the basolateral complex of the amygdala in AV-WT mice of the Paired group. The boxed areas in the left panel are enlarged in the middle and right panels.

C Representative examples of CaMKIIα-positive (upper panels), CaMKIIα-negative (middle panels) and GAD67-positive (bottom panels) neurons with Venus fluorescence in the LA.

D Stacked bar charts of the percentage of CaMKIIα-positive and -negative cells with Venus fluorescence (mean ± SEM). A few cells were undistinguishable whether they were CaMKIIα positive or not.

E Stacked bar charts of the percentage of GAD67-positive and -negative cells with Venus fluorescence (mean ± SEM).

 © 2021 The Authors

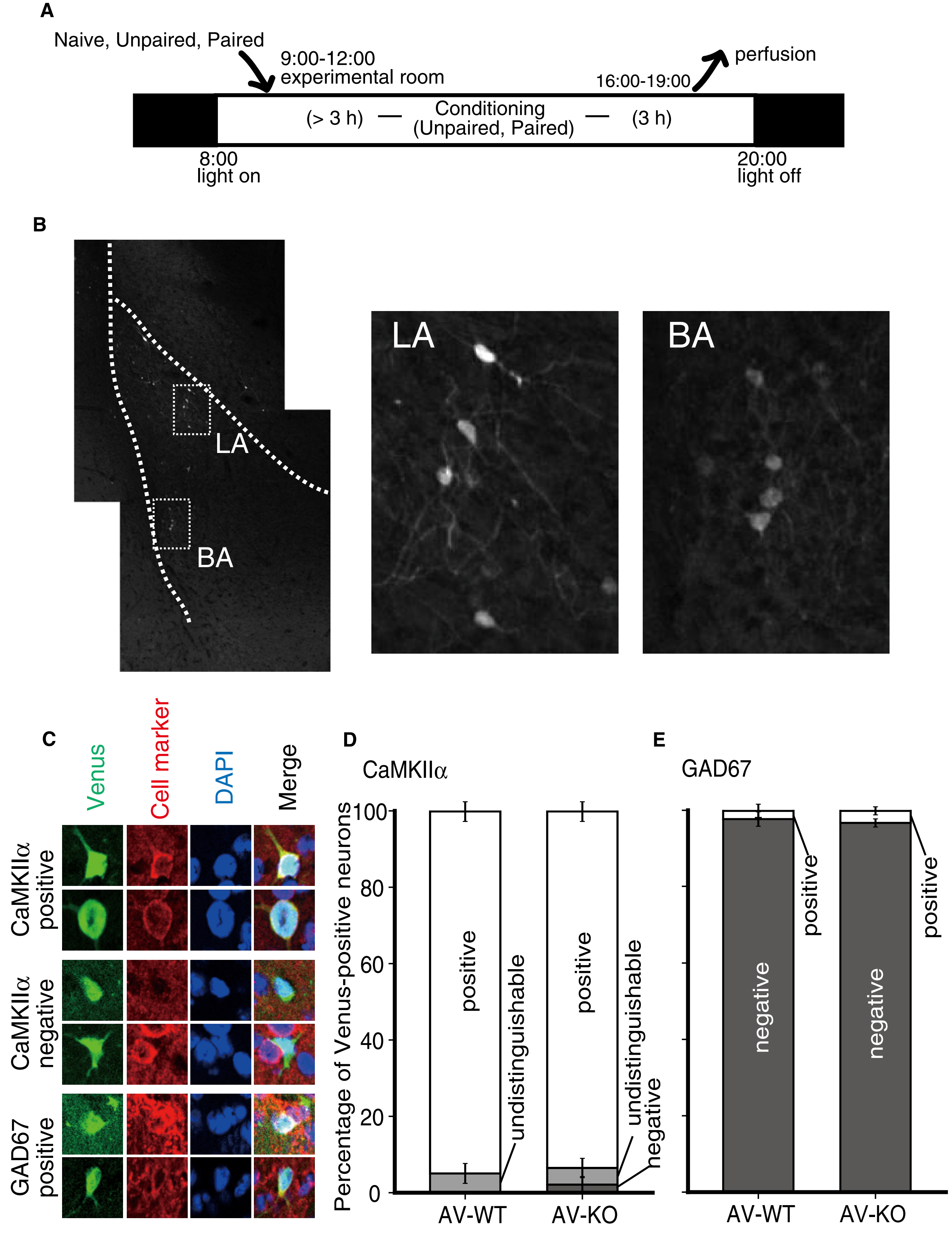
A
Naive, Unpaired, Paired
9:00-12:00
experimental room
perfusion
16:00-19:00
(> 3 h)
Conditioning
(Unpaired, Paired)
(3 h)
8:00
light on
20:00
light off
B
LA
BA
LA
BA
C
Venus
Cell marker
DAPI
Merge
CaMKIIα positive
CaMKIIα negative
GAD67 positive
D
CaMKIIα
Percentage of Venus-positive neurons
100
80
60
40
20
0
positive
undistinguishable
positive
undistinguishable
negative
AV-WT
AV-KO
E
GAD67
positive
positive
negative
negative
AV-WT
AV-KO

**Figure EV3.**

| Motif Found | Discovery/Enrichment Program | E-value | Known or similar motifs |
|---|---|---|---|
| 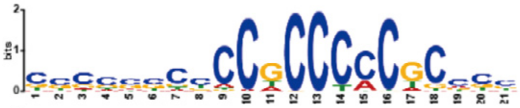 | MEME | $1.2 \times 10^{-492}$ | EGR1 (MA0162.2; MEME-Tomtom) |
| 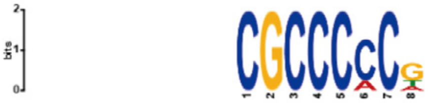 | DREME | $1.1 \times 10^{-146}$ | EGR1 (MA0162.2; DMEME-Tomtom) |
| 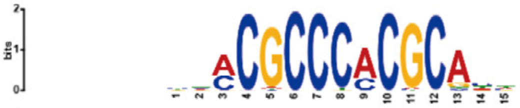 | CentriMo | $1.0 \times 10^{-123}$ | EGR3 (MA0732.1) |
| 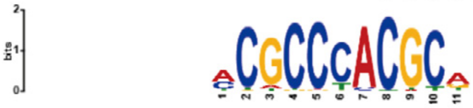 | CentriMo | $2.3 \times 10^{-108}$ | EGR2 (MA0472.2) |
| 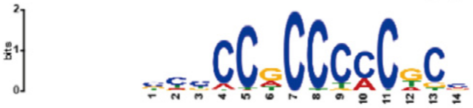 | CentriMo | $6.9 \times 10^{-102}$ | EGR1 (MA0162.2) |
| 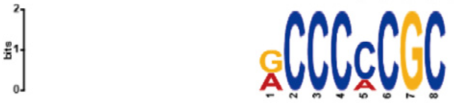 | CentriMo from DREME | $2.6 \times 10^{-93}$ | EGR1 (MA0162.2; DMEME-Tomtom) |
| 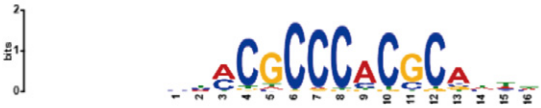 | CentriMo | $4.0 \times 10^{-73}$ | EGR4 (MA0733.1) |
| 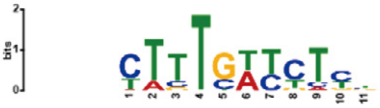 | MEME | $2.7 \times 10^{-68}$ | LEF (MA0768.1, MEME-Tomtom) |
| 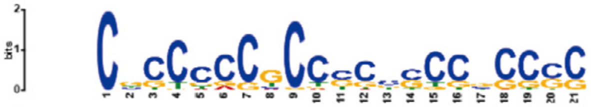 | CentriMo from MEME | $1.7 \times 10^{-48}$ | EGR1 (MA0162.2, MEME-Tomtom) |
| 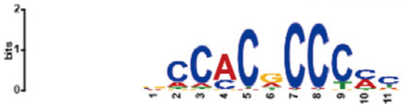 | CentriMo | $1.5 \times 10^{-41}$ | SP3 (MA0746.1) |

**Figure EV4. Top 10 motifs identified in the MEME-ChIP analysis of the LDB2-ChIP data.**

See the main text for the detail.


© 2021 The Authors

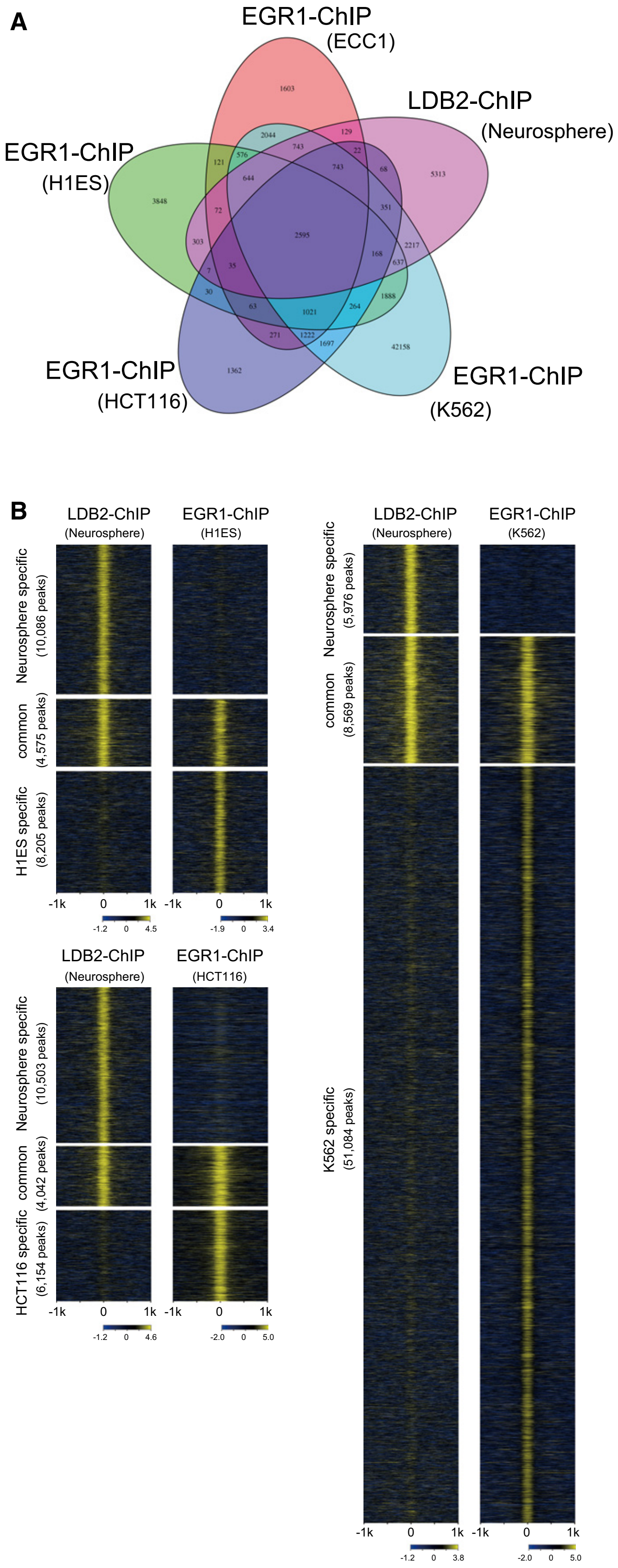


**Figure EV5. Overlap of LDB2 and EGR ChIP-seq peaks.**

A Venn diagram displaying the overlap of ChIP-seq peaks of LDB2 in neurosphere cells and ChIP-seq peaks of EGR1 in human cultured cell lines (ECC1, H1ES, HCT116, and K562).

B Genome-wide pattern of overlap between the ChIP-seq peaks of LDB2 in neurosphere cells and the ChIP-seq peaks of EGR1 in the cultured cell lines (H1ES, HCT116, and K562).

© 2021 The Authors
